# Image-Based Automated Width Measurement of Surface Cracking

**DOI:** 10.3390/s21227534

**Published:** 2021-11-12

**Authors:** Miguel Carrasco, Gerardo Araya-Letelier, Ramiro Velázquez, Paolo Visconti

**Affiliations:** 1 Facultad de Ingeniería y Ciencias, Universidad Adolfo Ibáñez, Av. Diagonal las Torres 2640, Santiago 7941169, Chile; miguel.carrasco@uai.cl; 2 Escuela de Construcción Civil, Pontificia Universidad Católica de Chile, Av. Vicuña Mackenna 4860, Macul, Santiago 7820436, Chile; gerardo.araya@uc.cl; 3 Facultad de Ingeniería, Universidad Panamericana, Av. Josemaría Escrivá de Balaguer 101, Aguascalientes 20296, Mexico; rvelazquez@up.edu.mx; 4 Department of Innovation Engineering, University of Salento, Via per Monteroni, 73100 Lecce, Italy

**Keywords:** surface cracks, crack characterization, infrastructure durability assessment

## Abstract

The detection of cracks is an important monitoring task in civil engineering infrastructure devoted to ensuring durability, structural safety, and integrity. It has been traditionally performed by visual inspection, and the measurement of crack width has been manually obtained with a crack-width comparator gauge (CWCG). Unfortunately, this technique is time-consuming, suffers from subjective judgement, and is error-prone due to the difficulty of ensuring a correct spatial measurement as the CWCG may not be correctly positioned in accordance with the crack orientation. Although algorithms for automatic crack detection have been developed, most of them have specifically focused on solving the segmentation problem through Deep Learning techniques failing to address the underlying problem: crack width evaluation, which is critical for the assessment of civil structures. This paper proposes a novel automated method for surface cracking width measurement based on digital image processing techniques. Our proposal consists of three stages: anisotropic smoothing, segmentation, and stabilized central points by k-means adjustment and allows the characterization of both crack width and curvature-related orientation. The method is validated by assessing the surface cracking of fiber-reinforced earthen construction materials. The preliminary results show that the proposal is robust, efficient, and highly accurate at estimating crack width in digital images. The method effectively discards false cracks and detects real ones as small as 0.15 mm width regardless of the lighting conditions.

## 1. Introduction

Over the last two decades, several techniques have been proposed to detect crack formation in materials and structures together with estimating its dimensions (width, length, and depth). The first image-based algorithms devoted to crack detection explored segmentation techniques targeting different types of civil engineering materials and infrastructures such as concrete, walls, and load testbeds. Recently, numerous advances have been made to develop techniques that consider the time evolution of loading with Digital Image Correlation (DIC) techniques [1,2] or hybrid methods that use a set of techniques and their combinations to improve the segmentation process [3,4,5]. In this way, it has been possible to perform experiments on different types of samples, which in some cases have allowed the evaluation of the use of different fiber types (e.g., synthetic, vegetal or animal) on different construction materials (e.g., mortar, concrete, and adobe) [6,7,8], as well as the study of the loading conditions over time on columns [9], beams and slabs [3], and walls [4] and the design of analytical models for estimating crack width [10] and surface analysis using machine learning techniques [11,12,13,14].

One of the main challenges for extracting and isolating the crack from the rest of the image is the implementation of segmentation algorithms. Research in this field has focused on two paradigms: (1) algorithms based on heuristic image processing and (2) algorithms based on Deep Learning (DL) tools.

The former includes techniques that evaluate the changes in the structure as a function of load [1,15,16,17], techniques that use one or more cameras for homography [18] or photogrammetry [15,19,20,21,22], and those that simulate a loading process and then analyze the material under pressure [19,23]. In general, the proposed solutions can be classified into techniques based on region growth [16,22], combinations of mathematical morphology and optimal threshold selection [5,24,25], techniques based on the Hessian matrix [26,27], and methods based on a sequence of partial filters that perform noise reduction as segmentation goes on [4,5,13]. The main advantage of techniques based on heuristic processing is that they can be directly applied to the samples without requiring any previous training. Nevertheless, they strongly depend on the type of material, implying that they might not always work properly.

The latter techniques have recently raised interest among researchers mainly because DL allows the implementation of robust solutions that can be applied in a wide variety of materials/structural elements such as paving, walls, and columns. In addition, it allows the use of different illumination conditions, orientation, distance, and objects [27,28,29,30]. DL-based algorithms rely on a learning process, which involves a previous labeling of images, so that the network can make a prediction through the learning process. Several types of network architectures have been explored in the literature [14,30,31]. The main difference across them is the generated output: images composed of blocks classified as failures [28,30], pixel-level binary segmentation [29,32,33,34], or a combination of both [34,35].

Both heuristic and DL paradigms exhibit different types of responses and require specific adjustments according to the exploited approach [31,36]. Surprisingly, despite the great advances in terms of segmentation, the literature presents limited progress with respect to crack width analysis [26]. Most related work found in the literature addresses crack width and length estimation without considering that width might change along with the material [27,29,33]. In these works, the middle section of the crack is indeed analyzed. However, no technique to measure the width as it progresses along the crack’s path is observed. An accurate image-based estimation of the crack width remains an open problem [9,24,31]. It is important to mention that different crack widths can be related to very different mechanical/durability effects within civil engineering materials [37]. Thus, an accurate crack width estimation is crucial since very fine cracks might be harmless and do not require further attention while wider cracks might require immediate attention and action. One of the main challenges in this topic is how to correctly measure the width while taking into consideration that the crack might exhibit a highly variable path, and therefore its width must be measured orthogonally to the path. Once this problem is solved, it becomes then relevant to consider the number of points to evaluate along the path.

**Figure 1:**
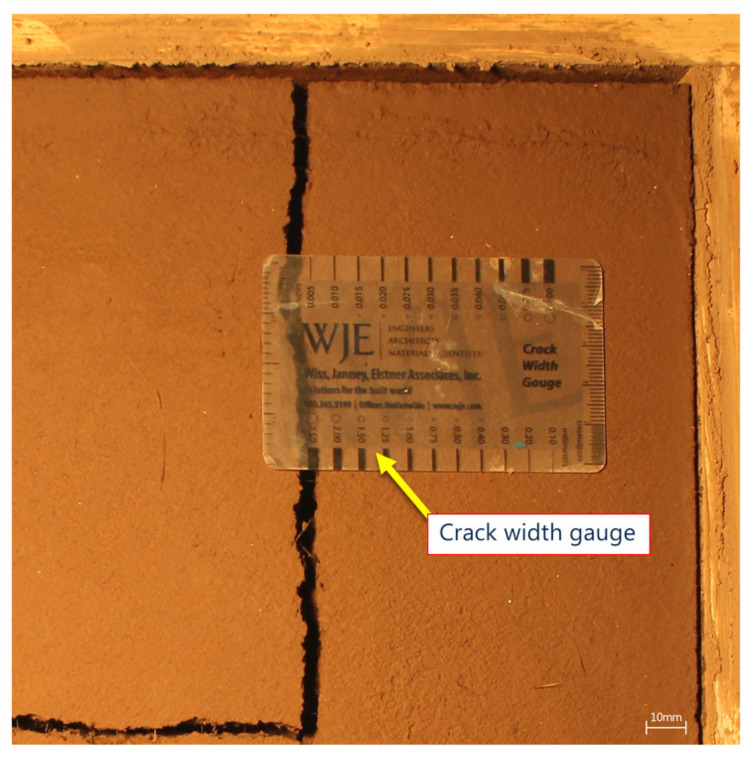
Crack-width comparator gauge (CWCG) for measuring the crack width; image resolution 14.7 pixels/mm.

Currently, the most popular method for crack measurement and characterization is by visual inspection using a crack-width comparator gauge (CWCG) [38], where the width of the crack is recorded manually over a distance interval using this CWCG (Figure 1). Although this technique allows for the rapid measurement of cracks in materials and structures, it is prone to serious errors: (i) the overlap between the measuring rule and the crack is not accurate as it depends on the inspector’s experience; (ii) depending on the orientation of the crack curvature, the crack measurement angle may not be accurate as it might not be tangentially measured; (iii) the measurement interval between two points might not be regular and may under or over represent the average crack width as cracks do not necessarily have a normal distribution [9] and (iv) the measurement is made by visual observation; therefore, it is subject to the inspector’s subjective measurement [39]. The above problems lead to the fact that the crack measurement can be underestimated or overestimated.

**Figure 2:**
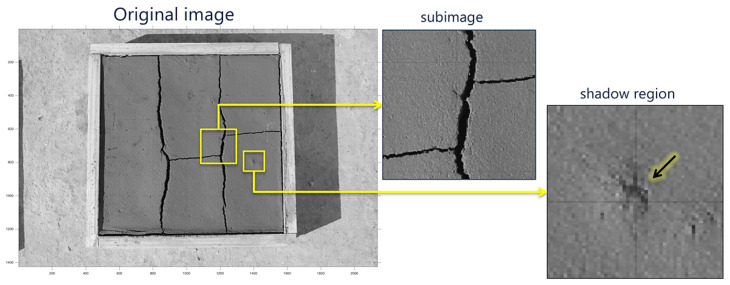
Test image with cracks and shadowed regions; original image area (500 mm × 500 mm), sub-image area (82 mm × 82 mm), resolution 11.5 pixel/mm, shadow region area (25 mm × 25 mm): Arrow: detail of a suspected fault region that is actually a shadow.

To avoid these errors, it is necessary to perform a non-contact measurement of the sample in such a way that there is no intervention on the crack to be measured. This requires the acquisition of images in which cracks are clearly exposed. However, there are some limitations inherent to the acquisition; for example, the incident angle of the light could generate shadows on the surface (Figure 2) that could lead to errors in crack detection. Depending on the material under analysis, the surface may generate false crack regions [31]. In this context, it is necessary to establish the correct position of the light source for the object to be analyzed. In general, the light should be positioned perpendicularly to the object to eliminate shadows and avoid false crack regions. This situation has been analyzed with different approaches, all of which are strongly dependent on the type of sample to be used. However, techniques using DL-based segmentation have proven to be more versatile for handling samples of different types of material, angles, and distances [30].

This paper proposes a novel image-based method for estimating crack width with a three-stage algorithm that effectively considers width variations along the crack’s path. By measuring the orientation angle that is normal to the crack curvature, the method allows for a more accurate estimation of the cracks present in the material or structure. Much of the work found in the literature has not focused on this problem. On the contrary, research has focused on solving the segmentation problem. Although segmentation is a highly relevant and complex problem, so is width estimation. Its correct measurement is essential to determine the actual condition of civil structures. Automating this process can be done after addressing the segmentation problem.

The rest of the paper is organized as follows: Section 2 describes the method for crack width estimation. Section 3 presents the results obtained with different sample materials. Finally, Section 4 concludes by summarizing the main concepts and results and giving perspectives on future work.

## 2. Method

**Figure 3:**
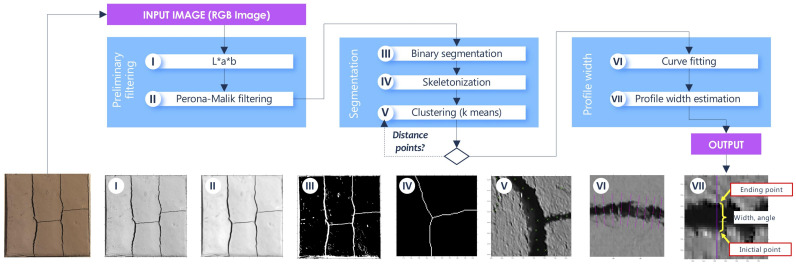
The proposed three-stage algorithm for crack width estimation. The partial results for each imagen of the algorithm are described in Section 2.1,Section 2.2,Section 2.3.

To determine the width of the cracks on an image, the proposed algorithm exploits three stages: (i) preliminary filtering; (ii) adaptive segmentation; and (iii) profile estimation (Figure 3). This section describes these stages. The following subsections describe each of these stages together with their corresponding intermediate steps.

### 2.1. Step I: Preliminary Filtering

One of the main challenges in the crack detection process is to reduce the false cracks, i.e., the regions that have visual characteristics similar to those of a real crack, such as shadows or elements embedded in the material, such as fibers, and digital noise. Therefore, the objective of the preliminary filtering stage is to reduce the number of false cracks through two filtering sub-stages: (i) color space change; and (ii) Perona–Malik filter.

The first sub-stage involves a color change using the well-known L*a*b color space [40]. This technique maximizes the luminance channel while allowing the attenuation of the effects due to shadows or light levels on the object. For this purpose, the algorithm performs an L*a*b channel transformation on the original RGB image. Only the L-channel of this transformation is retained.

The second sub-stage uses the filtering technique proposed by Perona and Malik [41,42,43]. This filter achieves noise reduction while preserving the fundamental structures of the image such as the edges. Let us consider a grayscale image *L* of dimensions (*n* × *m*) represented by gray values $f\left(x,y\right)\in \left({\mathbb{R}}^{2}\right)$ in positions (*x*, *y*). The proposed filter is defined by Equation (1):

(1) $$\partial_{t}L=div\left(c\left(\left\|\nabla L\right\|\right)\;\nabla L\right),\;with\;\partial_{t=0}L=L$$

where *div*(∙) represents the divergence operator, ∇ is the gradient operator, and *c*(∙) is a non-increasing smooth diffusion operator with *c*(0) = 1 and *c*(*s*)→0 with *s*→∞, which depends on the location of the point in the image.

**Figure 4:**
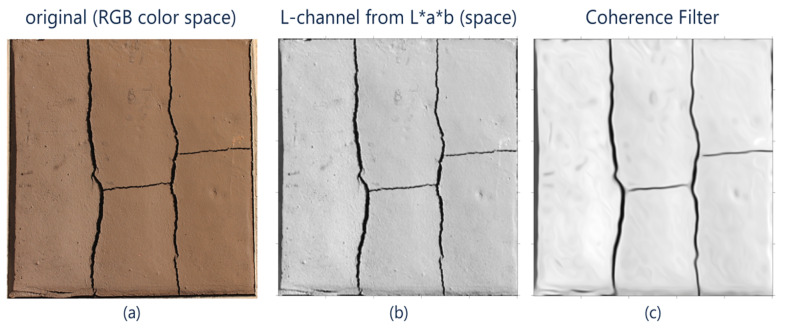
Preliminary filtering step: (**a**) original image captured with a Canon XTI Rebel 6i camera, (**b**) L-channel from the L*a*b space, and (**c**) coherence filter applied to the L-channel; sample area 500 mm × 500 mm.

According to variable *t*, this operation is iterative. To achieve a result, it requires a number of previously user-defined iterations. In our proposal, the number of iterations is a fixed parameter. Concerning the diffusion operator, different models can be found in the literature [41]. In this work, we implement the linear model proposed by Weickert [44] that uses a technique known as coherence filter [45]. Figure 4 shows a performance example of the filtering stage.

### 2.2. Step II: Binary Segmentation

Let us assume that the result of the previous stage stopped at time *t*. In addition, let us consider $W=\partial_{t}L$ as the image resulting from the coherence filter. Applying operator $⊓\left(\cdot \right)$ over matrix *W* generates a vector $w=⊓\left(W\right)$ of dimensions (*nm* × 1) (see Appendix A). The next procedure involves the search of threshold *δ*, which relates each value of vector *w* to a binary value, in other words, the process known as segmentation. To that end, let us further define vector *w* as a monotonically increasing function with values ordered through an order function $w^{+}=sort\left(w\right)$. The threshold’s value can be determined by Equation (2):

(2) $$\delta =\underset{i}{\underbrace{argmax}}\left(w^{+}\left(i\right)\left(nm-i\right)\right)\forall i=1,\ldots ,nm$$

**Figure 5:**
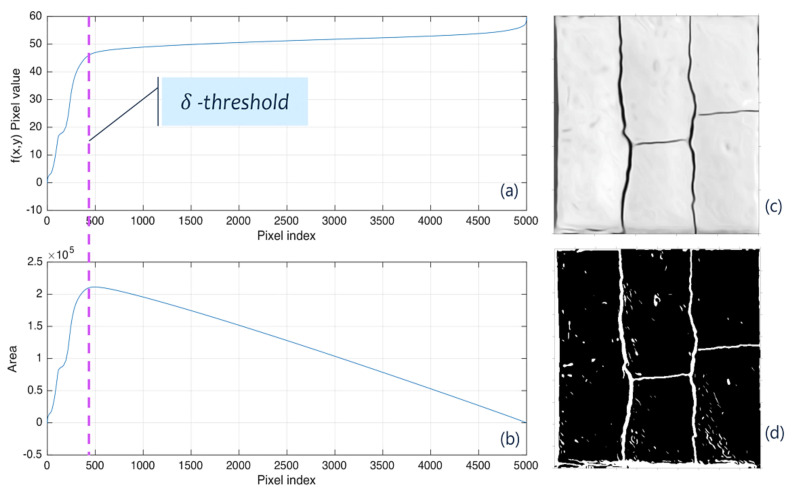
(**a**) Vector *w*^+^ in the original image, (**b**) the maximum index for Equation (2), (**c**) the coherence filter output, and (**d**) binary segmentation; sample area 500 mm × 500 mm.

Image segmentation can be defined as the process of relating all pixels in the image to a binary value $\forall f\left(x,y\right)>\delta \to Β\left(x,y\right)=1$, otherwise $Β\left(x,y\right)=0$, where $Β\left(x,y\right)$ is a matrix of dimensions (*n* × *m*) (Figure 5).

Once the binary edges have been determined, our proposal proceeds to determine the central position of the potential cracks. For this purpose, the method employs the skeleton algorithm based on the distance transformation process [46], which allows the simplification of the image to determine the centroids of each crack.

**Figure 6:**
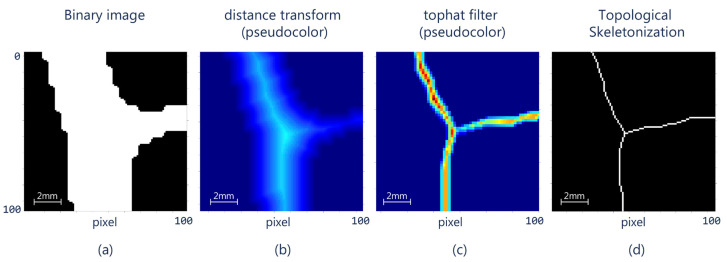
(**a**) A section of the binary image, (**b**) the Euclidean distance transform on the binary image (in pseudo-color), (**c**) application of the top-hat filter to the distance transform, and (**d**) topological skeleton applied to the top-hat image (zoom area 12.8 mm × 12.8 mm).

The distance transform is a measure of the distance between a pixel and its edge. The farther the pixel from the edge, the higher its value [24,47]. Subsequently, the method applies the top-hat filter in such a way that only the pixels with the maximum value remain stable. This procedure eases the application of the topological filter to find the skeleton of the structure (Figure 6). Even though it is possible to directly apply this last filter to the image shown in Figure 6a, the resulting skeleton would be incorrect since other regions that are not part of the central structure of the crack could appear given that the crack exhibits a greater width in other regions.

Let *S* be the binary matrix containing the crack skeleton (Figure 6d) and *M* a coordinate matrix satisfying Equation (3):

(3) $$M_{i}=\forall x\in \left[1,..,m\right]\wedge \forall y\in \left[1,..,n\right]\;\exists \;\left(x,y\right)\;|\;S\left(x,y\right)=1$$

Depending on the number of coordinates having the logical value 1, the number of indices may vary for each p-tuples. Since the pixels of the skeleton are neighbors to each other, one way to reduce the spacing is to determine a minimum distance *d_min_* corresponding to the minimum distance between a point and its neighbor.

The proposed procedure iterates the number of clusters of the k-means algorithm [48] until a certain distance between the centers is reached. Assume that $C=kmeans\left(M,\;k\right)$ is a subset of tuples of matrix *M*, i.e., C⊂M for *k* centroids. The purpose is to find distance *d_min_* according to *k* given in Equation (4):

(4) $$d_{min}>\underset{k}{\underbrace{argmin}}\;\left(\left\|C_{i}-C_{j}\right\|\right)\;\forall i,j\;|\;i\neq j\wedge \forall k\in {\mathbb{N}}^{+}$$

**Figure 7:**
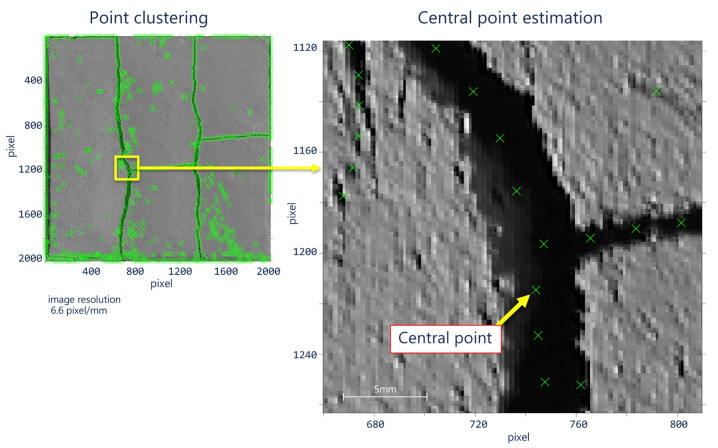
Central points after the neighbor spacing stabilization process; central point area equal to 28.3 mm × 28.4 mm.

Figure 7 illustrates this concept.

### 2.3. Step III. Profile Width

**Figure 8:**
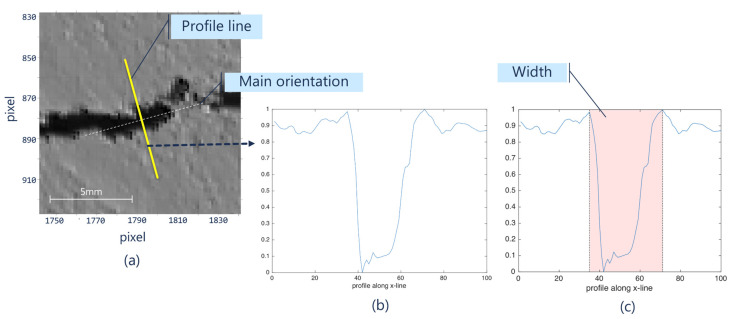
(**a**) Sub-region and line profile superimposed on the angle normal to the crack, (**b**) intensity levels of the profile, and (**c**) width estimation by clustering.

Once the central point and neighboring spacing have been determined in the image, the next step involves the calculation of an optimal orientation angle that is normal to the crack curvature at each point. For this purpose, we use a 40 × 40-pixel mask centered on each point described above, and over this point, we extract a binary sub-region, previously defined as matrix *B*. The central angle can be estimated as the angle of the major axis of an ellipse that fits the boundary of the binary region (details of this algorithm can be found in [49]). This ensures that the measurement of the ellipse’s width is correct and in accordance with the path and curvature of the crack (Figure 8a). Once both the angle and the position have been defined, the method proceeds to determine the width by superimposing a profile line greater than the width of the crack (Figure 8b).

As the cracks have a significantly different intensity level with respect to the background, it is possible to determine a cluster that identifies two groups of pixels {*background*, *crack*} through the *k*-means algorithm. Let *P_i_* be the *i*-th vector comprising the intensity levels of length *t*. The width of the crack can be calculated as (Equation (5)):

(5) $$\begin{array}{c} width\left(i\right)=\sum (kmeans\left(P_{i}\left(1,\;t/2\right)==class\_crack\right)+\\ (kmeans\left(P_{i}\left(\frac{t}{2}+1,t\right)==class\_crack\right) \end{array}$$

where *class crack* is previously defined as the lower intensity value of the two groups, and the values of the vector indices range from 1 to *t*.

## 3. Results

**Figure 9:**
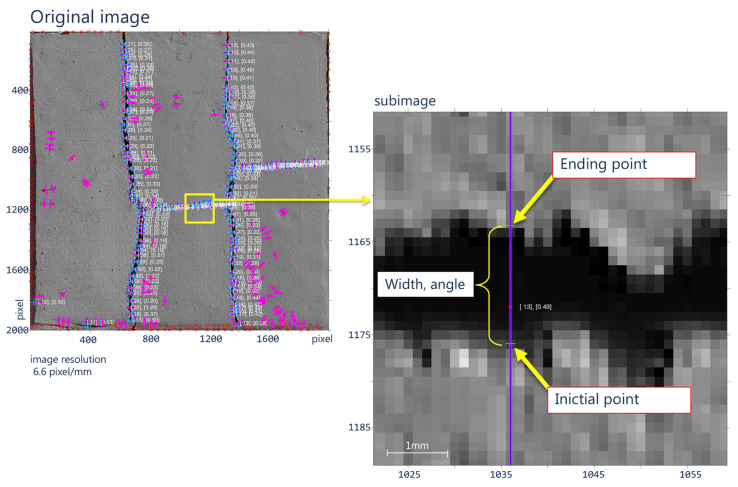
Plain mixture specimen with optimum calculation of the crack width and optimum angle of orientation according to the curvature.

The proposed method was evaluated on a set of earthen construction material (ECM) samples since this type of construction material is very susceptible to cracking [50,51] and, thus, different types of fibers (i.e., vegetal, synthetic, and animal) are used to reduce cracking and to compare this cracking reduction to an ECM sample without fibers (plain mixture). As previously explained, the algorithm searches for the optimal angle of orientation on the crack’s curvature according to a user-defined spacing for estimating its width and measurement angle, in the same way as a human operator would do with a crack monitor (Figure 9). Since each crack is locally analyzed, the computing time varies according to the number of cracks present in the sample.

**Figure 10:**
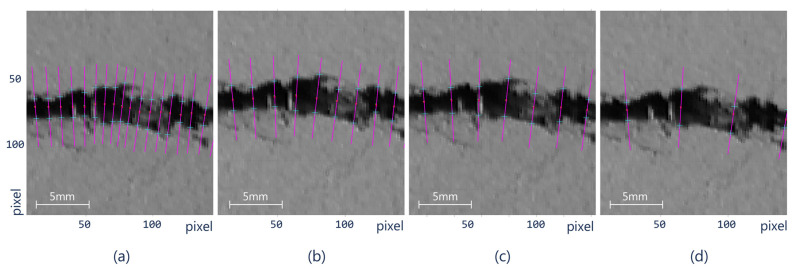
Control point spacing according to the k-means parameter *k*: (**a**) *k* = 10, *d_min_* = 8.5, *d_avg_* = 3.238 mm, (**b**) *k* = 15, *d_min_* = 13.5, *d_avg_* = 3.236, (**c**) *k* = 20, *d_min_* = 16.5, *d_avg_* = 3.364, and (**d**) *k* = 40, *d_min_* = 38, *d_avg_* = 3.158.

It is important to highlight that the number of width samples can be modified by the user implying that the resulting crack width will vary according to the number of samples (Figure 10). Due to sample variation, it is possible to estimate the error and the standard deviation. Therefore, it is possible to obtain a confidence interval for the measurement of the samples and to estimate the optimal number for such measurements. This fact is relevant since it allows obtaining a more accurate measurement compared to the current methods, which are limited in the number of crack width samples.

**Figure 11:**
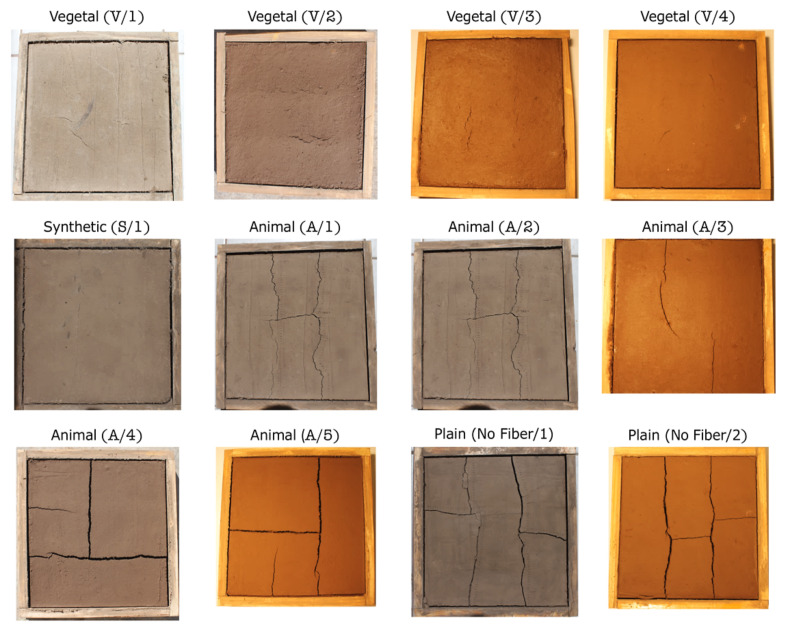
Dataset of samples generated from ECMs reinforced with different fiber types and a plain ECM. Samples were taken with two light sources: natural and artificial (area under analysis 500 mm × 500 mm).

The dataset used to validate the proposed method was generated from an ongoing study that analyzed the influence of the width of different fiber types (i.e., vegetal, synthetic, animal) in terms of the cracking reduction of ECMs that were also compared to a plain ECM (no fiber) (Figure 11). Depending on factors such as fiber type and dosages, it is possible to observe different cracking patterns, which generate a variable number of cracks, angles, and widths [51,52]. Additionally, to evaluate the method’s performance under different scenarios, samples were exposed to both natural and artificial lighting.

**Figure 12:**
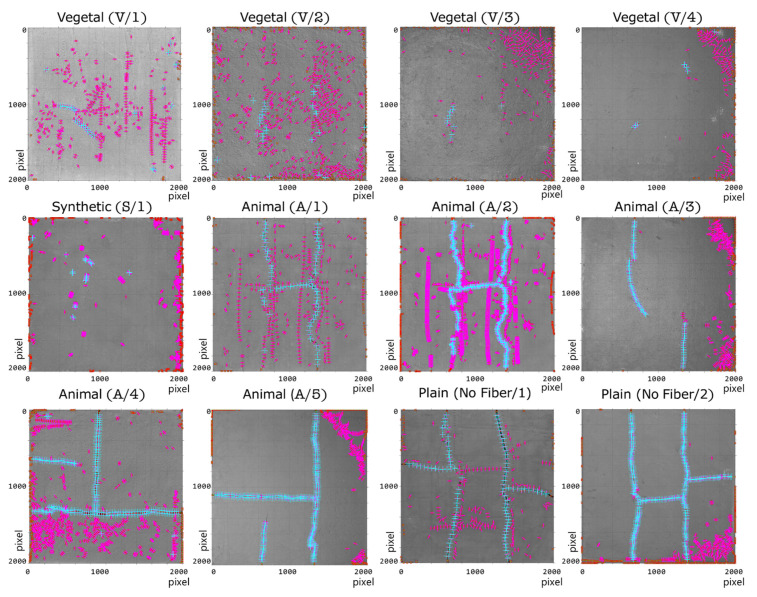
Results of the proposed method on the dataset of Figure 11 (area under analysis 500 mm × 500 mm).

Despite the different lighting conditions in the image dataset, the proposed method exhibits a stable behavior due to the extraction of the luminance channel from the L*a*b transformation, thus reducing the effect produced by the type of light source in the sample (see Figure 12).

**Figure 13:**
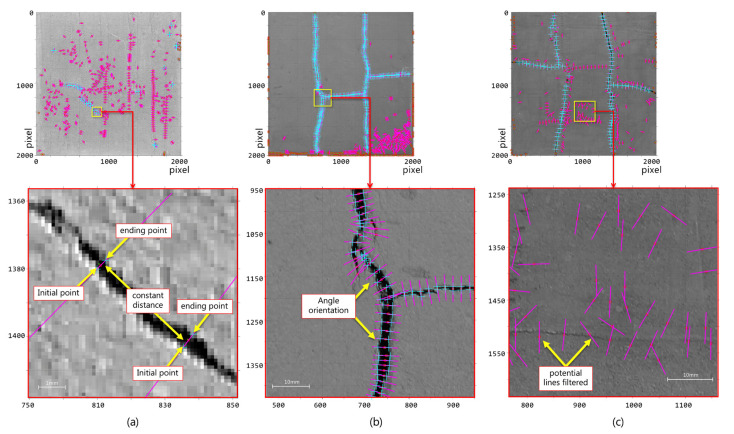
Zoom on the analyzed images (**a**), zoom area: 8.3 mm × 8.4 mm), (**b**), zoom area: 61.5 mm × 61.5 mm), and (**c**), zoom area: 51.3 mm × 51.3 mm).

On the other hand, in noisy areas generated by non-regular surfaces, the coherence filter allows a reduction in most of the structures that do not correspond to real cracks, thus maintaining their continuity and reducing the number of false alarms (Figure 13c). This effect is more noticeable in some samples of vegetal fibers (V/2 and V/3 fiber), for which surfaces exhibit irregular zones that could be erroneously segmented as cracks (Figure 13c). However, a possible undesired effect is that some measurement points can be filtered out when the crack width is very small (less than 0.15 mm).

In general, the results indicate that the crack samples, which exhibited different types of fibers and lighting, were correctly analyzed by the proposed method and that the width of the cracks was determined with high accuracy (Figure 13b). Furthermore, for those areas in which there is no relevant change between the surface and the background, the method discards the possible candidate regions (Figure 13c). This is achieved upon the analysis of the distance between the crack’s background and surface. The above concept is represented by purple (fuchsia) lines without the blue markers.

To statistically validate the proposed method, a manual width estimation process was performed on the images of the dataset. A total of 330 crack width measurements were extracted. For each sample, 30 crack width measurements were obtained taking into account the crack curvature. Each of the manual points was randomly selected with respect to the dispersion of the cracks in the samples. Both results, manual and automatic, were compared by means of a *t*-test with non-equivalent variance (Welch Test [53]). 

Table 1 summarizes the results obtained. Note that each sample comes from the same distribution according to the values obtained in Z-score and *p*-value for each of the samples (manual versus automatic). The most significant differences were found in those images with a low point extraction. In such images, a higher number of samples is necessary to make a valid comparison (Figure 11, sample V/4).

**Figure 14:**
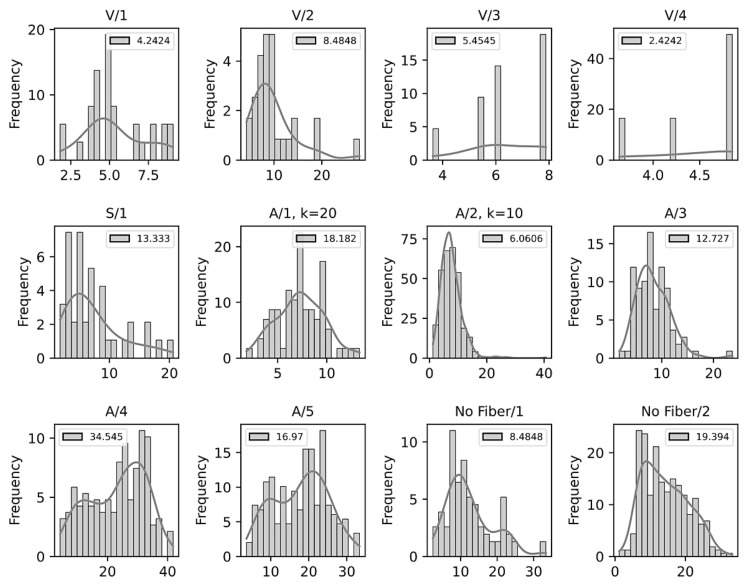
Frequency distribution of the measurements performed automatically.

According to the conditions defined in Table 1, the crack width distribution was evaluated to analyze its behavior (Figure 14). Recall that the method performs a point spacing between the set of measurements, thus ensuring that each measurement is completely unbiased in a parameterized way (parameter *k*). The differences in crack width across the samples are associated with a mechanical stress present in the interaction between the fiber and the mixture [50,52]. Still, it is possible to visualize zero, one, or more underlying distributions depending on the type of crack present in the sample.

We discuss the possible types of distributions below.

(1) No clear distribution: Samples V/3 and V/4 show this case. In these samples, it was possible to extract only a limited number of measurement points due to the small number of cracks present in the sample. Thus, it is not possible to associate the data with a specific distribution. This limitation of the method is a particular point for future improvement.
(2) Normal distribution: This phenomenon is present in samples with a regular cracking pattern. It is clearly observed in samples A/1, A/2, and A/3. Additionally, in sample A/2, the number of measurements was increased from 75 to 606 through parameter *k* (Figure 10). By increasing the number of measurements, it is possible to observe a normal behavior and a lower error in relation to the manual process (*p*-value 0.906 versus *p*-value 0.308).
(3) Bimodal distribution: This phenomenon can be clearly observed in samples No Fiber/1, A/4, and A/5 (Figure 13). These samples exhibit two types of cracks (coarse and fine), which have a vertical and/or horizontal cracking behavior. In some cases, a greater width was found in the horizontal cracks. However, it is worth mentioning that all the cracks have internal angles that can only be appreciated in images with a higher magnification (Figure 13a).

**Figure 15:**
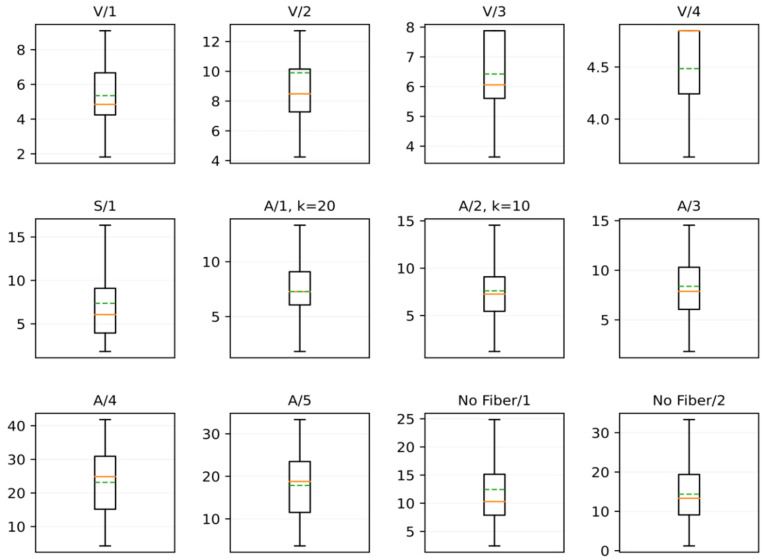
Boxplot of the measurements performed automatically.

The previous analysis is complemented with a boxplot (Figure 15). Overall, it can be observed that the cracks exhibit a variation of less than five pixels (0.75 mm) in the IQR range for each sample. The above does not apply to samples A/4 and A/5, in which the cracking pattern has a larger variation; wide open and closed crack widths can be found in the same sample with an approximate variation of 15 pixels (2.25 mm). It is important to note that the proposed method was evaluated on cracks with a width greater than one pixel (1 pixel = 0.15 mm); however, this relationship depends on the image resolution, which can be improved if the resolution of the sample images is increased. In addition, it can be observed that the median and mean in most of the analyzed samples are very close to each other, thus ensuring the numerical stability of the method by greatly reducing the number of false alarms (see Figure 14, median: green dotted line, mean: orange line). As previously discussed, the main variations can be found in samples V/3 and V/4 due to an insufficient number of measurements to define a distribution. In the rest of the distributions, the behavior is normal, either with univariate or with bivariate distributions due to the type of cracks present in the samples.

## 4. Conclusions

This paper has presented an efficient and highly accurate method for estimating crack width in digital images and this method can be easily applied to the cracking assessment of construction materials, such as mortar, concrete and adobe, which is an important task to evaluate in civil engineering materials and structures. The proposed method considers the orientation of the crack curvature and ensures that the measurement is unbiased and parameterized (i.e., at regular intervals) and was evaluated on a dataset of earthen construction material (ECM) samples that present samples reinforced with different fiber types (i.e., vegetal, synthetic, animal) as well as a plain sample (no fiber).

The dataset presents different crack widths that are within the range of 0.15 to 5 mm. Images were digitized using two types of light sources (natural and artificial) to evaluate the method’s performance in real situations (laboratory and external environment). For external environments, two additional lighting conditions were used: sunlight and afternoon sun to check the effect of shadows on the cracks.

The results show that there is no statistical difference between the manual and automated measurement. This represents a great advantage since the measurement time of the proposed method is at least 20 times shorter than that of the manual process. In addition, it was possible to obtain a robust confidence interval due to the larger number of measurement points compared to the manual method, which is tedious, time-consuming, prone to human error, and potentially biased. The method is independent to the type of light used, which facilitates the analysis of samples in outdoor environments.

The main difference between our method and DL-based techniques is that the latter focuses exclusively on the segmentation stage. It is true that segmentation is fundamental for crack detection, and our method acknowledges the importance of involving a segmentation stage. However, determining how best to estimate crack width remains an open problem with just a few works in the literature addressing this topic. Most of the methods already reported in the literature perform crack analysis by considering groups of pixels without any continuity. However, cracks exhibit a tortuous behavior, and it becomes essential to preserve their path along the material. Threshold-based algorithms directly applied on the image tend to break this continuity thus limiting any further analysis on the crack width. This limitation justifies the use of the Perona–Malik technique in our proposal which allows us to highlight the path’s continuity while reducing noise (Table 2, noise removal column).

Another major advantage of our method lies in the estimation of the actual crack width. Some works assume that width can be measured either horizontally or vertically and do not consider the crack’s motion or angle. A limited number of studies report on this analysis but do so without considering the crack as an object of variable width. This last concept is fundamental as, at the pixel level, the crack can be visualized as a group of pixels with tortuous motion, with width and angle variations according to the type of stress applied to the sample (Table 2, route tracing column).

Finally, width estimations must be performed on the whole sample at regular intervals, so that the measurement result is unbiased. Most of the previous studies do not take this point into consideration; they only analyze crack bodies, or they only perform an analysis at the pixel level and do not measure the real width of the cracks as suggested by the international building codes (Table 2, spatial width distribution).

The major limitation of the proposed method can be appreciated in samples with limited crack width, which considerably reduces the number of measurement samples. In these cases, in future work, an analysis of the type of crack should be defined prior to the definition of the number of measurements on each sample.

## Figures and Tables

**Table 1:** The method’s performance applied automatically and manually for 30 random points.

	Automatic Measurement			Manual Measurement (30 Points)		*t*-Test Comparison		Image Features		
Image Code	Count	Mean (Pixel)	std (Pixels)	Mean (Pixels)	std (Pixels)	Z-Score	*p*-Value	Light Type	*k*	Fiber Type
V/1	30	5.35	1.99	5.26	1.44	−0.25	0.801	Midday sun	20	Vegetal
V/2	30	9.90	4.95	9.55	2.04	−0.43	0.671	Midday sun	20	Vegetal
V/3	10	6.42	1.43	6.56	2.08	0.20	0.842	Light (3000 K)	10	Vegetal
V/4	5	4.48	0.54	4.32	1.11	−0.50	0.624	Light (3000 K)	20	Vegetal
S/1	39	7.37	4.72	6.21	4.65	−1.03	0.308	Afternoon Sun	20	Industrial
A/1	75	7.28	2.40	7.80	1.87	0.88	0.386	Afternoon Sun	20	Animal
A/2	606	7.61	3.72	7.80	1.87	0.11	0.906	Afternoon Sun	10	Animal
A/3	95	8.38	3.26	8.83	3.06	0.49	0.622	Light (3000 K)	20	Animal
A/4	192	23.15	9.39	22.71	8.22	−0.31	0.761	Midday sun	10	Animal
A/5	234	17.86	7.15	17.67	7.79	−0.38	0.699	Light (3000 K)	10	Animal
NoFiber/1	95	12.43	6.36	12.47	7.39	−0.39	0.694	Afternoon Sun	20	No Fiber
NoFiber/2	308	14.37	6.42	14.83	8.14	0.22	0.827	Light (3000 K)	20	No Fiber
TOTAL	1719 regions									

**Table 2:** Comparison with other techniques and their main features.

Technique	Noise Removal	Length Estimation	Route Tracing (Tortuosity)	Light Source Setup	Spatial Width Distribution	Ref
Edge distance + Linear fit	None (automatic threshold)	No	No	Yes	No	[15]
Percolation + Binarization	Percolation processing	No	No	No	No	[16]
Percolation + Neighbor boundary	Percolation processing	No	No	No	No	[22]
Skeletonization between two points	None (clumping process)	No	Yes	No	No	[23]
Digital image correlation (DIC)	None (automatic threshold)	Yes	No	Yes	No	[2]
Top-Hat + Otsu Binarization	Gaussian function-based spatial filter	No	No	No	No	[5]
Feature extraction and SVM algorithm	Steerable Filter	Yes	No	No	No	[4]
Genetic Algorithm	Multi-sequential image filter	Yes	Yes	No	No	[13]
Deep Learning	Fast-RCNN + TuFF	No	No	No	No	[29]
Hessian structure propagation	None	No	Yes	No	No	[27]
Deep Learning	YOLO	Yes	No	Yes	No	[33]
filtering + edge searching.	Frangi filtering	Yes	No	No	No	[26]
Bwdist transform + Arc Length	Morphological operations (Aperture)	Yes	Yes	Yes	No	[24]
M2GLD	Min-Max Gray Level Discrimination	No	No	No	No	[25]
Proposed technique	L*a*b + Coherence Filter	Yes	Yes	No	Yes	

## Data Availability

https://github.com/mlacarrasco/crack_width (accessed on 30 September 2021).

## Appendix A

Let *X* be a matrix of dimensions (*n* × *m*) and let *e_i_* be the i-th canonical basis vector of dimensions (*m* × 1), i.e., *e_i_* = [0, ⋯, 0, 1, 0, ⋯, 0] with a value of 1 at the *i*-th position and the rest with zeros. Operator $⊓\left(\cdot \right)$ can be defined as Equation (A1):

(A1) $$⊓\left(X\right)=\sum_{i=1}^{m}\left(e_{i}⊗I\right)Xe_{i}$$

where ⊗ represents the Kronecker product, *I* is the identity matrix of dimensions (*n* × *n*). The result of this operation for every matrix *X* of dimensions (*n* × *m*) is transformed into a vector of length (*nm* × 1). The inverse process, i.e., transforming a vector of length (*nm* × 1) into a matrix of dimensions (*nm ×* 1) can be defined by Equation (A2):

(A2) $$⊔\left(⊓(X)\right)=\sum_{i=1}^{m}\left(e_{i}⊗I\right)⊓(X)e_{i}^{T}=X$$

We will write $⊔\left(\cdot \right)$ as the vector-matrix transformation process, it being consistent with the number of existing elements in the vector and resulting in a matrix of dimensions (*n* × *m*).
